# Passive-Cooling Building Coating with Efficient Cooling Performance and Excellent Superhydrophobicity

**DOI:** 10.3390/ma16155232

**Published:** 2023-07-25

**Authors:** Xiaowei Yang, Defeng Yan, Yi Lu, Yulin Shang, Jing Sun, Jinlong Song

**Affiliations:** 1 State Key Laboratory of High-performance Precision Manufacturing, Dalian University of Technology, Dalian 116024, China; yangxiaowei1997@163.com (X.Y.); yandefeng1994@163.com (D.Y.); louis_13626240358@163.com (Y.L.); 15252504644@163.com (Y.S.); 2 Key Laboratory for Micro/Nano Technology and System of Liaoning Province, Dalian University of Technology, Dalian 116024, China

**Keywords:** passive-cooling building coating, superhydrophobic, anti-fouling, durability

## Abstract

Passive-cooling building materials can achieve cooling without external energy consumption, which is an energy-saving and environmentally friendly cooling method. However, the existing passive-cooling building materials have the limitations of high cost, complicated processes, and a toxic organic solvent, which hinders the passive-cooling technology applied in practical building. To overcome these limitations, we developed a facile, high-efficiency, non-toxic, and superhydrophobic passive-cooling building coating (SPCBC) with an efficient cooling capability and excellent durability that was composed of polydimethylsiloxane and SiO_2_. The fabricated SPCBC demonstrated a high reflectance and a high emittance, showing a superior cooling capability with a 14 °C temperature drop compared with a bare cement surface on a hot summer day. In addition, the SPCBC could not be wetted or contaminated by muddy water, corrosive aqueous solutions, or dust, which presented an excellent anti-fouling and self-cleaning capability. Moreover, the fabricated SPCBC could work outdoors for 30 days, withstand UV irradiation for 30 days, and resist accelerated aging for 100 h without any significant changes in the superhydrophobicity and the cooling capability, meaning that the SPCBC had an outstanding durability. This work provides a new method to facilitate passive-cooling technology to apply in practical building in hot weather regions of the world.

## 1. Introduction

Buildings, as places for people to live and work, are important to maintain a comfortable indoor temperature [1]. To maintain a comfortable indoor temperature in a building, people usually use air conditioning to lower the indoor temperature during the hot summer [2]. However, this cooling method consumes a large amount of electricity; for instance, the air conditioning for cooling represents 28% of the total electricity consumption in Hong Kong [3]. This method to lower the indoor temperature of buildings is not environmentally friendly and violates the green development concept [4,5,6]. Therefore, it is necessary to find a low-cost and environmentally friendly cooling method to achieve the effective cooling of building interiors.

Passive-cooling technology has proven that it is a low-cost, green cooling method to achieve indoor cooling for buildings without additional energy consumption [7,8,9,10]. However, the cooling capability of this method was not significant. To enhance the cooling capability, much research has been conducted, but it was still difficult to achieve a significant cooling capability until 2018 [11]. In that year, Yu et al. developed a passive-cooling material composed of a hierarchically porous polymer that had a significant cooling effect [11]. Since then, a series of porous or hierarchically porous polymers have been developed to achieve indoor cooling for buildings [12,13,14]. Nevertheless, these methods still had the problems of poor weather resistance and a susceptibility to contamination, which weaken the cooling capability [15,16]. To solve the aforementioned problems, a superhydrophobic surface was applied in passive-cooling technology to improve its contaminant resistance and durability; the fabrication methods could be mainly divided into the template method and the coating method [12,17,18,19,20,21,22,23,24]. In the template method, the polymer is pressed into a metal template with the rough structures, and then the superhydrophobic surface is obtained by etching the metal template using an acid such as an HCl aqueous solution, which is harmful to the environment [17,18]. In addition, the template method is also not conducive to the large-scale fabrication of a passive-cooling surface to be applied on a building’s exterior surface [19,20]. The coating method is usually conducted such that the polymer, nanoparticles, and bonding agent are added into the organic solvent; then, the coating solution can be obtained by mixing the aforementioned organic solvent [12,21]. The coating solution is brushed or sprayed on the substrate surface to obtain the passive-cooling surface. Although the coating method is promising for the large-scale fabrication of passive-cooling surfaces, the fabrication process is complicated, and the organic solvent usually used is toxic tetrahydrofuran, which seriously damages the health of the operators [22,23,24]. To promote the practical application of the passive-cooling building material, we have to find a new method to overcome the aforementioned serious limitations of the high cost, complicated process, and toxic organic solvent.

Here, we successfully overcame the limitations of the existing passive-cooling building materials and developed a facile, high-efficiency, and non-toxic passive-cooling building coating with superhydrophobicity and a high durability that was composed of polydimethylsiloxane (PDMS) and nano SiO_2_. We first measured the reflectance and the emittance of the fabricated superhydrophobic passive-cooling building coating (SPCBC). Then, the cooling capability of the SPCBC was measured on a sunny hot day and a cloudy hot day. In addition, the self-cleaning capability, anti-fouling capability, chemical stability capability, environmental corrosion resistance capability, UV corrosion resistance capability, and accelerated aging resistance capability were tested. Our work will hopefully facilitate the application of passive-cooling technology in practical building in hot weather regions of the world.

## 2. Materials and Methods

### 2.1. Materials

Polydimethylsiloxane (PDMS) and the curing agent were purchased from Dow Corning (Midland, MI, USA). Nano SiO_2_ particles (20 nm) and sub-micro SiO_2_ particles (200 nm) were purchased from Guihuang Trading Co., Ltd. (Xingtai, China). The hydrophilic polytetrafluoroethylene (PTFE) filter film was purchased from Chuangwei Filter Equipment Co., Ltd. (Jiaxing, China). Ethyl acetate was obtained from the Guanghua chemical reagent factory (Guangzhou, China). Commercial cooling film (CCF) with a thickness of 400 μm was purchased from SANYOU Co., Ltd. (Changzhou, China). Aluminum (Al) sheets with a size of 3 cm × 3 cm and a thickness of 1 mm were purchased from Shanxi Metal Co., Ltd. (Shenzhen, China).

### 2.2. Fabrication of the Superhydrophobic Passive-Cooling Building Coating

**Figure 1:**
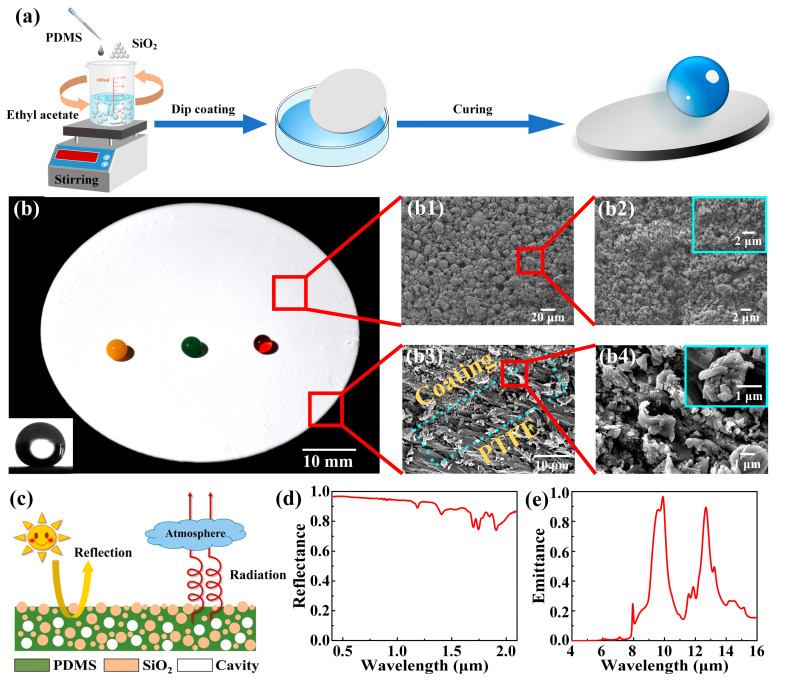
Fabrication processes, surface morphology, optical properties, and cooling performance of the SPCF. (**a**) The fabrication process of the SPCF. (**b**) The optical image of the SPCF. (**b1**–**b4**) The SEM images of the SPCF. (**c**) The working principle of the SPCBC. (**d**) The reflectance of the SPCBC. (**e**) The emittance of the SPCBC.

The main fabrication processes of the superhydrophobic passive-cooling building coating (SPCBC) are shown in Figure 1a. Firstly, the PDMS, the curing agent, the SiO_2_ with 20 nm particles, and the SiO_2_ with 200 nm particles were added together into the ethyl acetate at a mass ratio of 10:1:5:5:120 [22]. Then, the mixture was stirred for 3 h with a magnetic stirrer (MS300, China) to obtain a uniformly dispersed suspension (Figures S1 and S2). In addition, we also investigated the influence of adding different nano SiO_2_ particle sizes on the cooling performance, as shown in Figure S3. To facilitate the characterization of this SPCBC, we immersed the purchased PTFE film into the prepared suspension for 5 min. After that, the film was placed in a fume cupboard for about 15 min to volatilize the ethyl acetate. Finally, a superhydrophobic passive-cooling film (SPCF) was obtained after drying in an oven at 80 °C for 2 h.

### 2.3. Sample Characterization

The surface morphology of the samples was observed by an emission scanning electron microscope (SEM, JSM7900F, Tokyo, Japan), and the element compositions were characterized by an energy dispersive spectroscope (EDS, JSM7900F, Tokyo, Japan). The reflectance of the samples in the 0.3 μm–2.5 μm bands was measured using a UV–Vis–NIR spectrophotometer (Lambda 1050+, Waltham, WLM, USA). The infrared emissivity was obtained by a Fourier Transform Infrared spectrometer (FTIR, VERTEX 80, Billerica, USA) with a test range of 2.5 μm–25 μm. The water contact angle (CA) and the rolling angle (RA) were measured at room temperature by an optical contact angle meter (SL200KS, Boston, BSN, USA) using a 5 μL water droplet, and three points were taken on the surface of each sample for testing, after which the average value was taken and used as the experimental value [25,26,27]. All optical images were recorded by a digital camera (DSC-RX10M3, SONY, Tokyo, Japan).

### 2.4. Cooling Performance Test

The cooling performance of the SPCF was obtained by comparing the temperature difference between an Al sheet with the SPCF and an Al sheet without any coating. A multi-channel temperature monitor (DC5508U, Zhongshan, China) was used to detect the temperature of the Al sheet [28]. The light intensity was measured using a solar optical power meter (SM206-SOLAR, Shenzhen, China). The cooling performance test of the SPCF included the cooling of the Al sheet, the cooling of the building model, and the cooling of cement.

The cooling of the Al sheet: Three experimental groups were set up which were an Al sheet without any coating, an Al sheet with a commercial cooling film, and an Al sheet with the SPCF. Then, the Al sheets were placed in a self-made test device, and the temperature of Al sheets was detected by the temperature monitor outdoors.

The cooling of the building model: One building model was covered with the SPCF, while the other was untreated. The size and material of the two building models were exactly the same (10.5 cm × 5.5 cm × 10 cm and a weight of about 45 g). The probes of the temperature monitor were inserted into the same position inside the building model, and detected the temperature inside the building model in real time. The cooling performance test was conducted on a hot sunny day and a hot cloudy day, respectively.

The cooling of cement: Two cement blocks with a diameter of 50 mm and a thickness of 10 mm were fabricated, one of which was covered with the SPCF and the other was not covered with anything. The probes of a multi-channel temperature monitor were placed on the top and the bottom of the block, respectively. The real-time temperature of the top and bottom of the two cement blocks was tested outdoors at the same time, and their temperature difference was calculated to characterize the cooling performance of the SPCF on the cement.

### 2.5. Versatility Test of the Superhydrophobic Passive-Cooling Building Coating

The versatility test of the SPCBC included the self-cleaning test, anti-fouling test, chemical stability test, environmental corrosion test, UV corrosion resistance test, and accelerated aging test.

Self-cleaning test: The chalk dust was used to simulate dust and was evenly spread on the SPCF. Then, the water droplets were dropped on the SPCF and rolled off the SPCF. The self-cleaning performance of the SPCF was evaluated by observing and recording whether the chalk dust remained on the surface [29].

Anti-fouling test: The muddy water was poured on the SPCF and the CCF surface with an inclination angle of 10°. Then, the cooling performances of two cooling films were tested by the temperature monitor to evaluate the anti-fouling ability [30].

Chemical stability test: Aqueous solutions of different pH values (pH = 0 to pH = 14) were prepared with HCl (dyed green), NaCl (dyed yellow), and NaOH (dyed red). The samples were placed on inclined plates with an angle of 10° and aqueous solution droplets with different pH rolled off the SPCF. The surface was observed and recorded for residual dyed droplets to evaluate its chemical stability [31,32].

Environmental corrosion resistance test: Passive-cooling building materials mainly work outdoors, and environmental corrosion resistance is an important index to test its actual working performance. The SPCF was placed outdoors and fixed on a plate, which was directly exposed to the air with the sunlight, wind, and rain. The experiment period lasted for 30 days, during which there were the sunny days and the rainy days. The contact angles and rolling angles of the SPCF were tested every five days. After 30 days, the cooling performance was tested outdoors to evaluate whether the SPCF still had the cooling capacity [33].

UV corrosion resistance test: The UV test was conducted in an enclosed environmental chamber maintained with a temperature of 25 °C, and a UV lamp with a wavelength of 365 nm was placed 20 cm away from the upper surface of the SPCF for 30 days to test its UV corrosion resistance. The changes in its wettability were recorded every 5 days by measuring the contact angles and the rolling angles. The cooling performance was tested after 30 days to evaluate whether the SPCF still had a cooling capacity [34].

Accelerated aging resistance test: An SPCF was placed in an oven with a temperature of 70 °C for 100 h. The changes in contact angles and rolling angles were recorded every 25 h to characterize the surface wettability of the SPCF, and after 100 h, the sample was tested outdoors to evaluate whether the SPCF still had a cooling capacity. This test was referred to the national standard of China, viz., GB/T 7141-2008 [35].

## 3. Results

### 3.1. Surface Morphology, Optical Properties, and Cooling Performance of the SPCF

The fabrication processes of the superhydrophobic passive-cooling film (SPCF) are shown in Figure 1a. It can be seen that the fabrication method of the passive-cooling building coating does not require the expensive equipment, which means it can be easily used for fabrication. The optical image of the SPCF is shown in Figure 1b. The static contact angle of water droplet on the film is 160°, showing an excellent superhydrophobicity which causes the water droplet to present a spherical shape on the film and quickly roll away from the tilted SPCF (Video S1). We then observe the microstructures of the SPCF by a scanning electron microscope, as shown in Figure 1(b1–b4). From the SEM images, it can be seen that the SiO_2_ particles generated an agglomeration phenomenon on the film surface. These agglomerated SiO_2_ particles made the film surface have micro-nano structures, which not only increased the light reflection ability of the passive-cooling coating, but also was a necessary condition to fabricate a superhydrophobic surface [36,37,38]. In addition, the SEM image of the film section shows that the SPCBC and the PTFE substrate are tight, which means that the SPCBC is difficult to peel off from the substrate, as shown in Figure 1(b3,b4) and Figure S4. Moreover, we also characterized the element compositions of the SPCBC and found that no F element was detected on the SPCBC, indicating that the SPCBC was green and non-toxic (Figure S5). Relevant literatures showed that the sunlight at the 0.3 μm–2.5 μm bands played a major function in heating the object, and the heat of the object could spread out to outer space in the form of the electromagnetic wave at the bands of 8 μm–13 μm [39,40,41]. Therefore, if the SPCBC has a high reflectance at the bands of 0.3 μm–2.5 μm and a high emittance at the bands of 8 μm–13 μm, it can hopefully be used for the building cooling, as shown in Figure 1c. We then measured the optical spectrum of the SPCBC in the solar and infrared radiation regions. The SPCBC exhibited a high reflectance that reduced the heat transfer from the sunlight. In the bands of 8 μm–13 μm, the SPCBC exhibited a high emittance that increased radiative heat transfer to the outer space.

**Figure 2:**
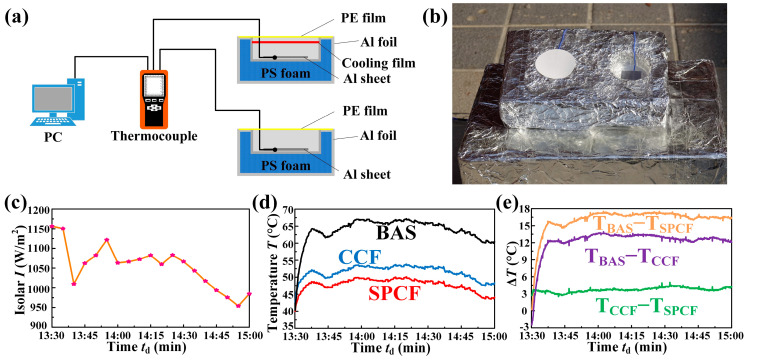
Cooling performance of the SPCF. (**a**) Schematic illustration of the test device. (**b**) Optical image of the actual test device. (**c**) Detailed solar intensity during the test. (**d**) Real-time temperature of the bare Al sheet, Al sheet covered by the CCF, and Al sheet covered by the SPCF. (**e**) Temperature differences of the BAS and the SPCF, the BAS and the CCF, and the CCF and the SPCF, respectively.

The cooling performance of the SPCF was tested to investigate whether the SPCBC had a cooling capacity in Dalian, China (38.8 N, 121.5 E, average altitude: 60 m) on 2022.5.31, which was conducted by the self-made experimental device. The experimental device is shown in Figure 2a. For comparison, two control groups were set up, which were a bare Al sheet without any cooling film (BAS) and an Al sheet covered with a commercial cooling film (CCF), respectively. To minimize the effect of heat conduction, the Al sheets and the different cooling films were placed in the cavities of a Polystyrene (PS) foam box with wrapping a layer of Al foil. Moreover, a layer of low-density polyethylene film (LDPE) was covered on the top of the PS foam box to prevent the heat convection generated by the air flow. The size of the Al sheets was absolutely identical, and the thermocouples were placed at the same positions of the Al sheets. The actual experimental device is shown in Figure 2b. We conducted the cooling performance of the SPCF on a sunny summer day with the solar intensity (I) between 950 W/m^2^ and 1200 W/m^2^ and the relative humidity (RH) between 15 and 25%, as shown in Figure 2c. Under the solar illumination for 8 min, the average temperature of the Al sheet covered with the SPCF was 48 °C, which is significantly lower than that of the BAS with 65 °C and achieveed the temperature drop Δ*T* = 17 °C, as shown in Figure 2d,e. It is worth noting that the temperature difference of the SPCF with Δ*T* = 17 °C was also higher than that of CCF with Δ*T* = 12 °C, which indicates that the SPCF has a superior cooling capability compared with the CCF, as shown in Figure 2d,e.

### 3.2. Cooling Performance of the SPCF on the Building Model and the Cement

**Figure 3:**
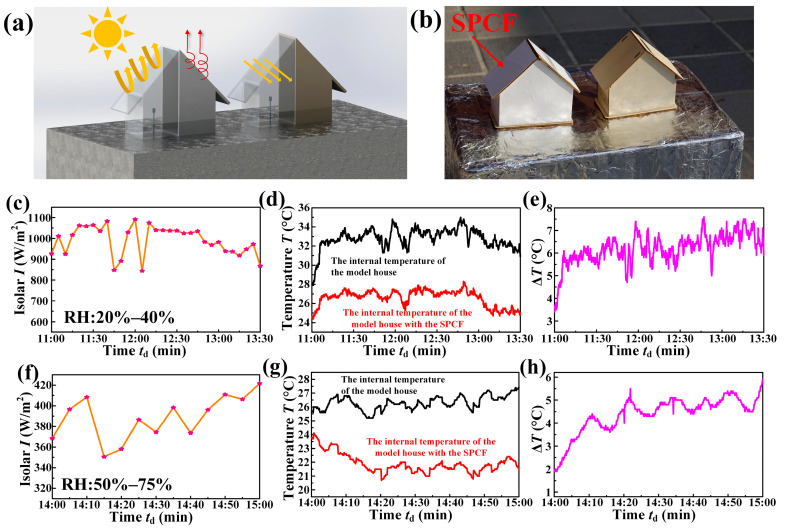
Practical application of the SPCF. (**a**) Test schematic diagram of the building models. (**b**) The experiment setup in the outdoor environment. (**c**) Solar intensity on the sunny day. (**d**) Temperature variation of the building model without any shading operation and building model with the SPCF on the sunny day. (**e**) Temperature difference of the SPCF group and the control group on the sunny day. (**f**) Solar intensity on the cloudy day. (**g**) Temperature variation of the building model without any shading operation and building model with the SPCF on the cloudy day. (**h**) Temperature difference of the SPCF group and the control group on the cloudy day.

Since the SPCF had a better cooling performance than the BAS and the CCF, we then studied whether the SPCF also had an excellent cooling performance on the actual outdoor wooden building model. We built two wooden building models on an open roof for comparative test, one of which was covered with the SPCF, while the other one was a bare building, as shown in Figure 3a. Regarding the actual environment, both of the buildings were directly exposed to sunlight without any shading operation, as shown in the Figure 3b. We conducted the actual building cooling performance of the SPCF on a sunny day from 11:00 to 13:30 (2022.06.19) with the relative humidity between 20 and 40% and the solar intensity between 850 W/m^2^ and 1100 W/m^2^ (Figure 3c), which was the hottest period of the day. The inside temperature of the two buildings was detected and recorded by the temperature detectors. It could be obviously seen that the inside temperature of the building without any cooling film was approximately equal to the environment temperature (Figure 3d). Interestingly, the average inside temperature of the building with the SPCF is about 27 °C, which is about 6 °C lower than that of the bare building with 31 °C, as shown in Figure 3d,e. The reasons for the temperature difference are that the building with the SPCF reflects part of the sunlight, and the heat of the building can radiate to the sky by the “atmospheric window”, rather than the bare building heats up quickly under the sunlight, resulting in the increasing inside temperature of the building. In the hot summer, even on the cloudy day, the inside temperature of the building still makes people feel uncomfortable. We tested the cooling performance of the SPCF on a cloudy day (2022.5.10) with the relative humidity between 50 and 70% and the solar intensity between 350 W/m^2^ and 420 W/m^2^ (Figure 3f). The average inside temperature of the building with the SPCF was about 21.5 °C, which was lower than the average inside temperature of the bare building with 26 °C (Figure 3g), achieving the temperature difference of about 4.5 °C (Figure 3g). Therefore, the SPCF has excellent cooling performance on the wooden building, which applies not only to the hot sunny days but also to the hot overcast days.

**Figure 4:**
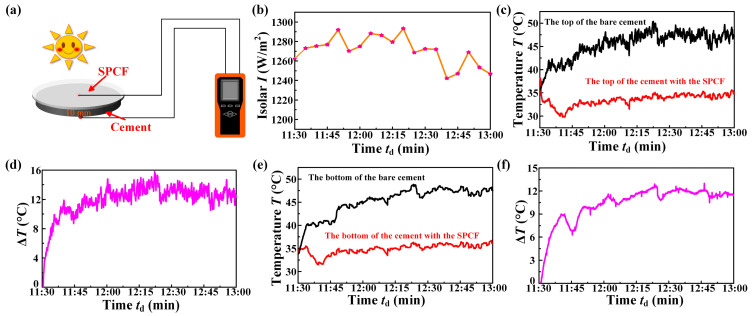
Cooling capability test of the SPCF on the cement. (**a**) Test schematic diagram of the cooling performance measurement. (**b**) Solar intensity during the experiment. (**c**) Temperature variation of the cement top without any cooling film and the cement top with the SPCF. (**d**) Temperature difference of the cement top with the SPCF and the bare cement top. (**e**) Temperature variation of the cement bottom without any cooling film and the cement bottom with the SPCF. (**f**) Temperature difference of the cement bottom with the SPCF and the bare cement bottom.

Cement is a commonly used material for the building exterior wall, and if the SPCF covers on the cement in the hot summer, it is possible to achieve temperature regulation without energy consumption by a simple operation [42,43]. The schematic diagram of the experimental setup is shown in Figure 4a. We conducted the cooling performance of the SPCF on a sunny summer day with the solar intensity between 1240 W/m^2^ and 1295 W/m^2^, as shown in Figure 4b. Interestingly, the top temperature of the cement block with the SPCF was significantly lower than that of the cement block without any cooling film (Figure 4c). The temperature difference of the cement top with the SPCF and the bare cement top was about 14 °C (Figure 4d). The bottom temperature of the cement block with the SPCF was also significantly lower than that of the cement block without any cooling film (Figure 4e). The temperature difference of the cement bottom with the SPCF and the bare cement top was about 12 °C (Figure 4f). It can be concluded that even with the cement, which is a practical building material, the SPCF still shows excellent cooling performance, which is important for achieving passive cooling in practical buildings.

### 3.3. Versatility of the SPCF

**Figure 5:**
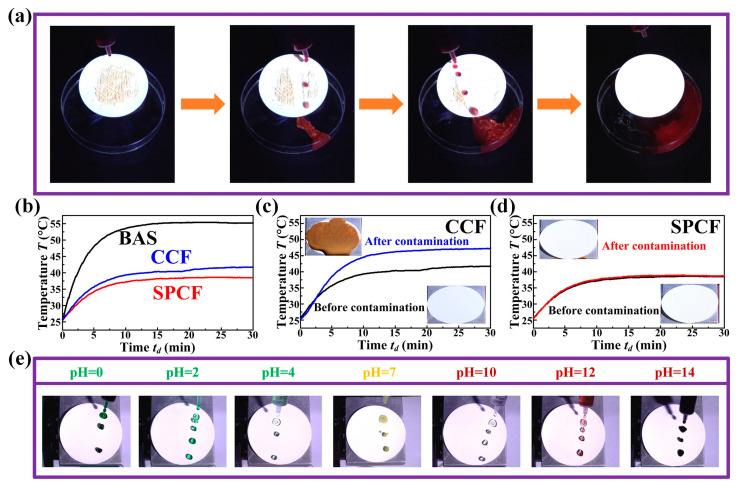
Anti-fouling property and chemical resistance tests. (**a**) Anti-fouling test of the SPCF. (**b**) Temperature measurement of three samples before muddy water pouring. (**c**) Cooling performance of the CCF before and after contamination. (**d**) Cooling performance of the SPCF before and after contamination. (**e**) Chemical resistance to droplets with different pH values.

Since the cooling materials are often used on the exterior of buildings, the dust covers the cooling material surface, resulting in the degradation of the cooling performance [44,45]. We used the chalk dust to simulate the dust particles and spread the chalk dust evenly on the SPCF surface. It can be seen that the dyed red water easily rolls on the SPCF surface and takes away the chalk dust without the immersion or contamination of the surface, resulting in that the initial SPCF with the contaminant is cleaned to the clean SPCF, as shown in Figure 5a and Video S2. In addition, the muddy water may also contact the cooling materials on the building and contaminate the cooling material surface, which affects the cooling efficiency. Therefore, we tested the cooling performance of the CCF and the SPCF before and after the muddy water pouring. Firstly, the temperatures of the bare Al sheet, the Al sheet covered with the CCF, and the Al sheet covered with the SPCF were monitored and recorded under a simulated solar light source with the solar intensity about 1000 W/m^2^ before the muddy water pouring, as shown in Figure 5b. We then poured the muddy water on the CCF and the SPCF surfaces with an inclination angle of 10°. It was obvious that a large amount of contaminants was left on the CCF surface, and the average temperature of the Al sheet covered with the CCF increased from 40 °C to 47 °C after pouring the muddy water (Figure 5c). On the contrary, the SPCF stayed clean and the cooling capacity of the SPCF did not show any significant change after pouring the muddy water, which was because the muddy water could easily roll off the SPCF surface compared with the CCF, showing the excellent anti-fouling property of the SPCF (Figure 5d and Video S3).

Since the exterior surfaces of building may be subject to the chemical corrosion such as the acidic solution and the alkaline solution, the acid and alkali resistance of the SPCF are also an important feature [46,47]. The HCl aqueous solution (dyed green), the 3.5 wt.% NaCl aqueous solution (dyed yellow), and the NaOH aqueous solution (dyed red) were prepared to test the acid and alkali resistance of the SPCF. The droplets with different pH from 0 to 14 were dripped on the SPCF surface and could easily roll off the surface without any residue, as shown in Figure 5e and Video S4. To further investigate the chemical stability of the SPCF, the SPCF was immersed in the HCl aqueous solution with pH = 5 and NaOH aqueous solution with pH = 13 for 20 min; then, the water droplet still easily rolled off the SPCF surface, as shown in Figures S6 and S7 and Videos S5 and S6. The aforementioned experiments showed that the fabricated SPCF has excellent chemical stability, which should be attributed to its superhydrophobicity, preventing the entrance of the aqueous solution into the interior of the SPCF.

**Figure 6:**
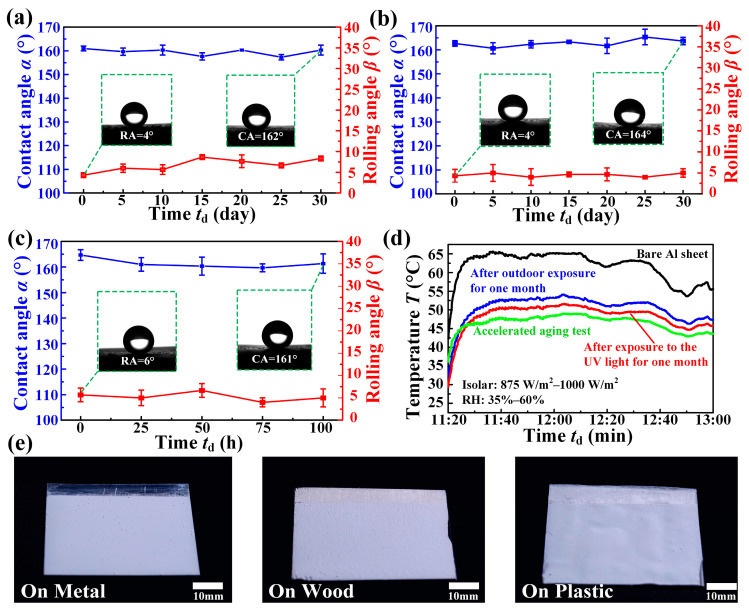
Durability test of the passive-cooling film. (**a**) Changes in CAs and RAs of the film under the outdoor condition for one month. (**b**) Changes in CAs and RAs exposed to the UV light for one month. (**c**) Changes in CAs and RAs in 100 h accelerated aging test. (**d**) The cooling performance after the durability test. (**e**) Coatings on different substrate materials.

In addition, we conducted experiments such as the natural environment corrosion, UV light corrosion, and accelerated aging to test whether the SPCF had weather resistance. It can be seen that there is no significant change in the water contact angles in the natural environment test of 30 days, and the water rolling angles remained below 10°, as shown in Figure 6a. During the UV corrosion and accelerated aging test, the water contact angles and the water rolling angles remained unchanged after the continuous UV corrosion of 30 days and the accelerated aging of 100 h, as shown in Figure 6b,c.

We then tested the cooling performance of the SPCF under the outdoor with the solar intensity between 875 W/m^2^ and 1000 W/m^2^ and the relative humidity between 35 and 60% after the natural environment test of 30 days, continuous UV corrosion of 30 days, and the accelerated aging of 100 h. Although the cooling capacity of the SPCF has slightly decreased due to the soiling with non-polar organic pollutants (e.g., soot particles, aerosols), it still has good cooling capacity with the maximum of 16 °C and the minimum of 13.5 °C after the durability test, as shown in Figure 6d. In addition, the coating used to fabricate the SPCF can be easily applied to a different substrate such as metal, wood, and plastic, which greatly expands its application range (Figure 6e). In addition, the SPCF can be dried not only in the oven but also at room temperature in the fabrication processes.

## 4. Conclusions

In this study, we successfully overcame the limitations of the passive-cooling building material and proposed a new method with high durability, superior cooling performance, and superhydrophobicity by fabricating a superhydrophobic passive-cooling building coating (SPCBC). The microscopic morphology was observed using SEM, and the optical properties were tested using FTIR and UV–Vis–NIR. A series of tests were designed and carried out to test its actual cooling effect, hydrophobic performance, and weathering durability performance, and the results showed that it had excellent overall performance. From the experiments, we found that the cooling effect of the samples prepared by using two particle sizes of SiO_2_ at the same time was better than that of the samples using only one particle size. Meanwhile, the rich microscopic rough structures and pores formed on the surface are beneficial to enhance the hydrophobic properties and reflectance of the film. Therefore, it is necessary to design special micro-nano structures on the surface in the subsequent research of passive-cooling technology.

## Data Availability

Not applicable.

## Supplementary Materials

The following supporting information can be downloaded at: https://www.mdpi.com/article/10.3390/ma16155232/s1. Figure S1. Image of suspension for the superhydrophobic passive-cooling building coating (SPCBC). Figure S2. The viscosity of the synthetized suspension used to fabricate the SPCBC. Figure S3. Cooling performance of the passive-cooling film fabricated by different particle size. Figure S4. The results of the adhesion test using the tape test. Figure S5. The main element compositions of the SPCBC. Figure S6. 20 min acid solution immersion experiment. Figure S7. 20 min basic solution immersion experiment. Video S1. Hydrophobicity test. Video S2. Anti-fouling test. Video S3. Anti-muddy water scouring test. Video S4. Corrosion resistance test. Video S5. Acid resistant solution test. Video S6. Basic resistant solution test.
