# Supplementary material for: Passive-Cooling Building Coating with Efficient Cooling Performance and Excellent Superhydrophobicity
Source: Materials (Basel). 2023 Jul 25;16(15):5232. doi: 10.3390/ma16155232 (PMC10419716; doi:10.3390/ma16155232)
Supplement: Supplementary file 1 [file materials-16-05232-s001.zip › Supplementary Materials.pdf]

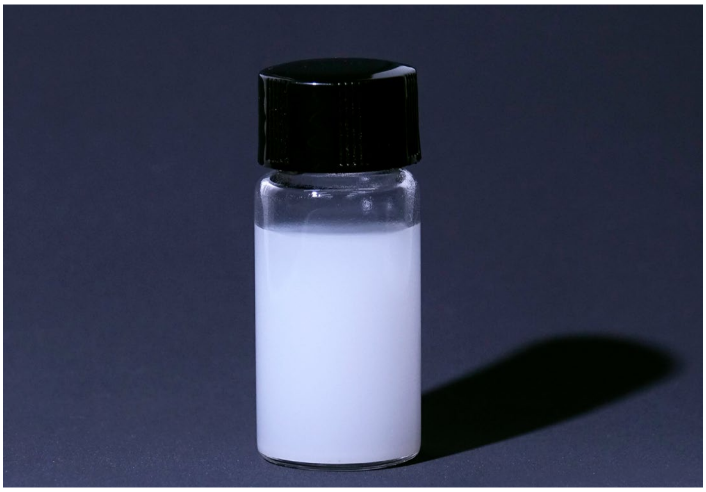

Figure S1. Image of suspension for the superhydrophobic passive-cooling building coating (SPCBC).

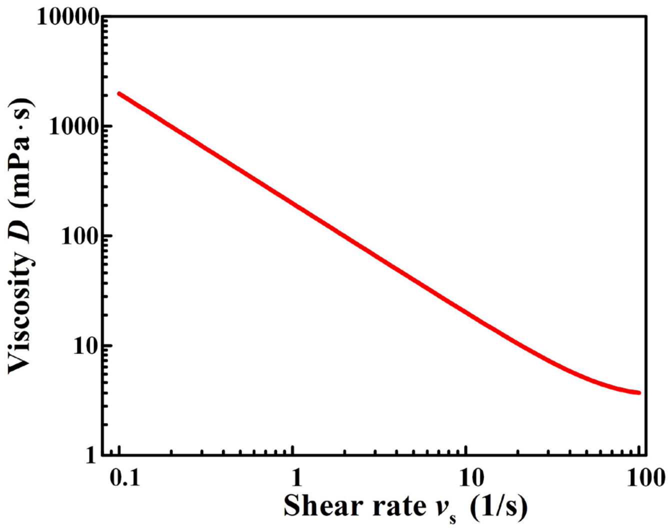

Figure S2. The viscosity of the synthetized suspension used to fabricate the SPCBC.

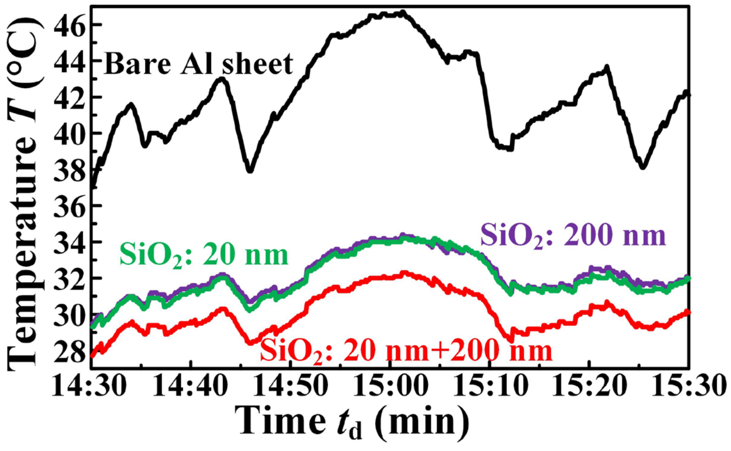

Figure S3. Cooling performance of the passive-cooling film fabricated by different particle size. Different particle

sizes of SiO_2 were selected to fabricate passive-cooling film with the same addition amount. The added particles were 20 nm, 200 nm, and a mixture of the two sizes. The cooling performance test was the same as the one used in the text, and the test diagram was shown in Figure S2. It could be seen that the passive-cooling films fabricated with different particle sizes of SiO_2 all had an obvious cooling effect. There was no significant difference in the cooling performance of the passive-cooling films fabricated using 20 nm (green line) and 200 nm (purple line) SiO_2 particles, while the passive-cooling films fabricated by mixing 20 nm and 200 nm (red line) SiO_2 particles had significantly better cooling performance. After several experimental tests, it could be found that the best cooling ability was achieved when the ratio of 20 nm particles to 200 nm particles was controlled at 1:1. In this experiment, the coating of the total solid mass with the 20 nm SiO_2 and 200 nm SiO_2 was same as that with only one type of particle.

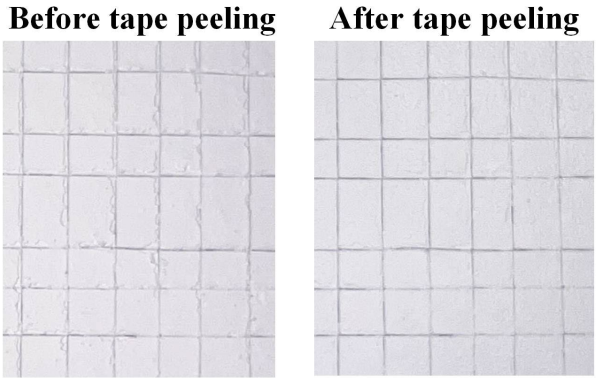

Figure S4. The results of the adhesion test using the tape test. The adhesion test was conducted based on the standard ASTM D3359-17. It could be seen from the Figure S4 that the coating still adhered well after the tape peeling test, indicating that it had good adhesion ability.

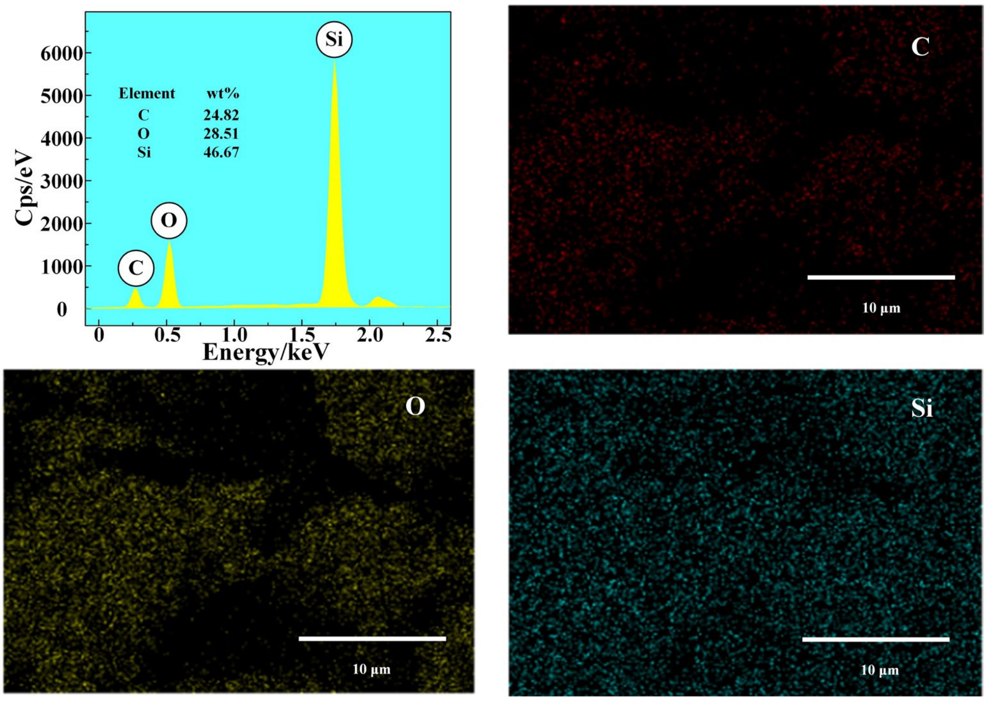

Figure S5. The main element compositions of the SPCBC, including C, O and Si, and the specific distribution was shown in the figure. Compared with the hydrophobic materials containing F element, the fabricated passive-cooling film was green and non-toxic as there was no F element at all.

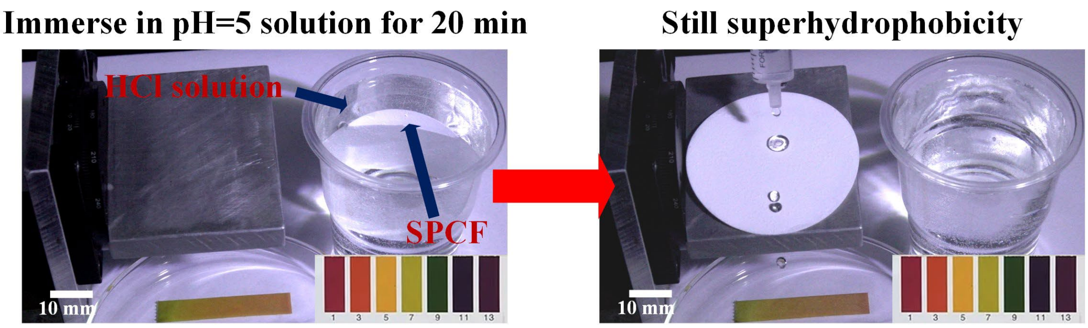

Figure S6. 20 min acid solution immersion experiment. An acidic solution with pH=5 was prepared using the 36.5 wt.% HCl solution. The fabricated SPCF was immersed in the acidic solution for 20 min, and removed on a tilted platform with an angle of 10°. Then, the water droplets were dropped on the SPCF surface and quickly rolled off the surface without any residue, showing the excellent corrosion resistance.

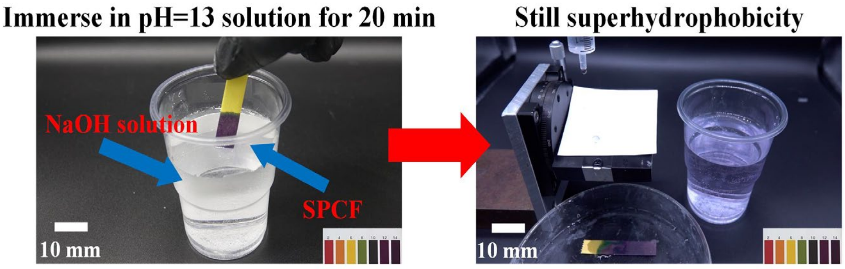

Figure S7. 20 min basic solution immersion experiment. The fabricated SPCF was immersed in a solution of pH=13 prepared with NaOH for 20 min, and removed on a tilted platform with an angle of 10°. Then, the water droplets were dropped on the SPCF surface and quickly rolled off the surface without any residue, showing the

excellent corrosion resistance. We have added the test results in the Video S6 to the supplementary materials.

Video S1.

Hydrophobicity test. The red dyed droplets were dropped on the surface of both samples. There was no visible residue on the SPCF surface, while the PTFE surface was absolutely saturated and a large amount of red solution was left on the surface.

Video S2.

Anti-fouling test. The chalk dust was used to simulate dust, which was evenly sprinkled on the surface of the SPCF. The droplet took away the dust in the process of rolling off the surface, and there was no obvious dust residue on the surface of the SPCF.

Video S3.

Anti-muddy water scouring test. The muddy water was splashed on the surface of the SPCF and the commercial cooling film (CCF) respectively, and there was no obvious residue on the surface of the SPCF, while a large amount of muddy water remained on the surface of the CCF.

Video S4.

Corrosion resistance test. The SPCF was placed on a platform with an inclination angle of 10°, and droplets of dyed liquids with different pH (pH=0 to pH=14) were dropped on the surface of the SPCF, and all droplets rolled off smoothly on its surface.

Video S5.

Acid resistant solution test. The SPCF was immersed in HCl solution with pH=5 for 20 min and then removed out. The superhydrophobicity was not affected as droplets dropped on the surface rolled off smoothly after the SPCF immersion.

Video S6.

Basic resistant solution test. The SPCF was immersed in NaOH solution with pH=13 for 20 min and then removed out. The superhydrophobicity was not affected as droplets dropped on the surface rolled off smoothly after the SPCF immersion.
